# A FPGA Implementation of the CAR-FAC Cochlear Model

**DOI:** 10.3389/fnins.2018.00198

**Published:** 2018-04-10

**Authors:** Ying Xu, Chetan S. Thakur, Ram K. Singh, Tara Julia Hamilton, Runchun M. Wang, André van Schaik

**Affiliations:** MARCS Institute, Western Sydney University, Sydney, NSW, Australia

**Keywords:** neuromorphic engineering, electronic cochlea, basilar membrane, inner hair cell, outer hair cell, automatic gain control, medial olivocochlear efferent, FPGAs

## Abstract

This paper presents a digital implementation of the Cascade of Asymmetric Resonators with Fast-Acting Compression (CAR-FAC) cochlear model. The CAR part simulates the basilar membrane's (BM) response to sound. The FAC part models the outer hair cell (OHC), the inner hair cell (IHC), and the medial olivocochlear efferent system functions. The FAC feeds back to the CAR by moving the poles and zeros of the CAR resonators automatically. We have implemented a 70-section, 44.1 kHz sampling rate CAR-FAC system on an Altera Cyclone V Field Programmable Gate Array (FPGA) with 18% ALM utilization by using time-multiplexing and pipeline parallelizing techniques and present measurement results here. The fully digital reconfigurable CAR-FAC system is stable, scalable, easy to use, and provides an excellent input stage to more complex machine hearing tasks such as sound localization, sound segregation, speech recognition, and so on.

## Introduction

The human auditory system is superior to any machine-hearing system in efficiency of perceiving sound. As the input structure for the auditory pathway, the tonotopically-organized cochlea decomposes, converts and amplifies sound waves nonlinearly into electrical signals, and delivers the results to the nervous system. The cochlea is characterized by a remarkably wide dynamic range (0-120 dB SPL) (Fettiplace and Hackney, 2006), and a high frequency selectivity (~3 Hz at the characteristic frequency of 1 kHz; Glasberg and Moore, 1990). Over the past decades, efforts have been made to engineer a hearing machine that is able to emulate the function and efficiency of the human auditory system. As a first step toward this target, cochlear models have been proposed, developed, and implemented in a number of ways with a varying degree of complexities.

### Auditory filter models

Cochlear models can be divided into two classes: transmission-lines (TL) and auditory filterbanks (Duifhuis, 2004). The TL models represent the cochlea partition as a coupled mass-spring-damper system to model wave propagation on the Basilar Membrane (BM) (Zweig et al., 1976). TL models are faithful to the physiology and are accurate in simulating wave propagation on the BM. However, they are more computationally challenging as they have complicated differential equations in the time domain (Altoè and Pulkki, 2014).

Auditory filterbank models use either parallel or cascade filters to model wave propagation on the BM. Parallel filterbank models use independent filters, such as rounded-exponential (roex) filters (Glasberg et al., 1984), the gammatone filter family (including gammachirp; Patterson et al., 2003), or pole-zero filters (Lyon et al., 2010), that connect to a single input signal in parallel. Cascade filterbank models, for example the CAR-FAC model (Lyon, 2017) or biophysical models of (Liu and Neely, 2010; Saremi and Stenfelt, 2013), use a cascade of filters instead.

Parallel filterbank models are mostly concerned with reproducing the observed mechanical and pay little attention to the biological structure of the cochlea. For example, Wang et al. implemented a parallel ultra-steep roll-off filter model on a 0.35μm CMOS chip (Wang et al., 2015), and Yang et al. implemented a parallel source-follower-based bandpass filterbank on a 0.18 μm CMOS analog IC (Yang et al., 2016). Some parallel filterbank models include an automatic gain control (AGC) mechanism to model some couplings between channels. For example, Yang et al. implemented a parallel filter bank of 4th-order one-zero gammatone filters (OZGF) with across channels AGC on a 0.35 μm CMOS chip (Yang et al., 2015). Another parallel form, the 2-D parallel filterbank, models the fluid within the cochlear duct as well as the BM taking both the longitudinal and vertical wave propagation into account. Examples of silicon cochleae of 2-D models include (van Schaik and Fragniere, 2001; Hamilton et al., 2008; Nouri et al., 2015).

Cascade filterbank models take advantage of the way sound propagates in the forward direction as traveling waves in the cochlea. In the cascade of filters, each filter stage models a segment of the nonuniform distributed wave system and its output becomes the input of the next section (Lyon, 1998). The cascade form thus provides a natural model of coupling in the forward direction. For example, Chan et al. implemented a 2nd-order low pass filter with address event interface (Chan et al., 2007), Liu et al. implemented a cascade 64-stage model on a 0.35μm CMOS chip (Liu et al., 2014), Thakur et al. implemented a CAR model on a FPGA (Thakur et al., 2014), and Jimenez-Fernandez et al. implemented a cascade spike band pass filer model on a FPGA (Jimenez-Fernandez et al., 2016). For some cascade filterbank models, such as Lyon's pole-zero filter cascade (PZFC) model and CAR-FAC model, an AGC feedback loop is included to model some couplings between channels in both directions. We describe the hardware implementation of the CAR-FAC model in this paper.

### Cochlea nonlinearity

**Figure 1:**
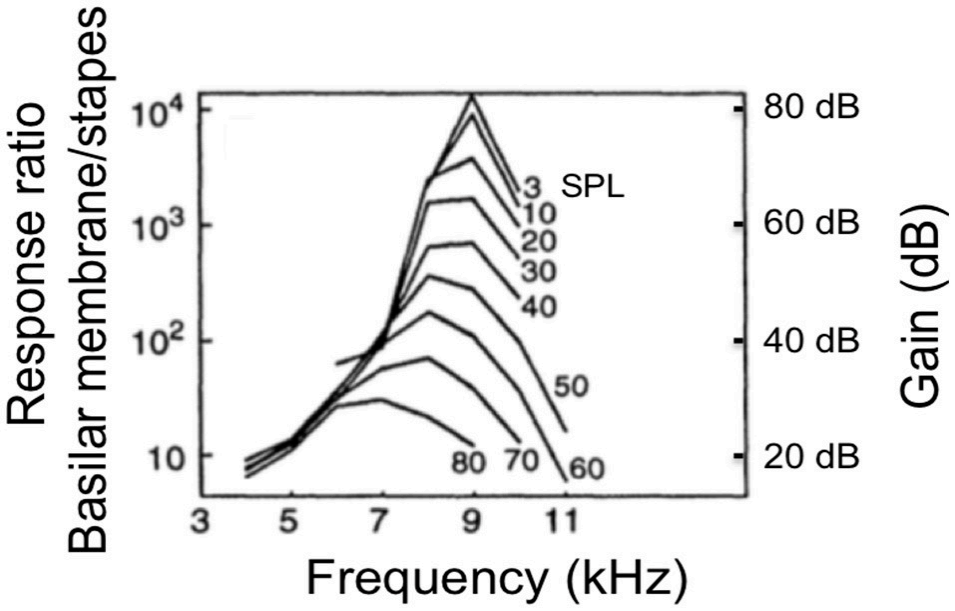
The frequency response measured from a chinchilla cochlea for various levels input strength measured in dB of sound pressure level (SPL) adapted from (Ruggero, 1992). The gain is measured by the BM displacement (or velocity) relative to the stapes motion.

The biological cochlea is a causal, active, and nonlinear system. Figure 1 shows the nonlinearity and frequency tuning measured from a biological cochlea for various sound pressure levels measured in dB SPL adapted from (Ruggero, 1992). The gain is measured by the BM displacement (or velocity) relative to the stapes motion. In the biological cochlea, responses at frequencies near the characteristic frequency (CF) (9 kHz) vary nonlinearly with input level. Additionally, the responses show steeper high-frequency roll-off slope at lower SPLs, and the peak gain shifts toward lower frequencies with increasing input level.

In auditory filterbank models, the nonlinearities can be described as linear filters with parameters depending on signal level. For example, the parallel and cascade gammachirp filter models (PrlGC and CasGC) (Irino and Patterson, 2001; Unoki et al., 2006), the all-pole gammatone filter (APGF) models and PZFC models (Lyon, 1997; Katsiamis et al., 2007) show a forward compressive nonlinear response via the movement of the poles and/or zeros. For AGC-based models, the output level is fed back to modify filter parameters, to result in a compressive input-output function (Lyon, 2011). Such a feedback nonlinearity mechanism is inspired by the OHCs function of the mammalian cochlea (Kim, 1986). The PZFC analog cochlear model (Lyon and Mead, 1988) and the CAR-FAC model (Lyon, 2017) are such examples.

### Motivations

The CAR-FAC model is a digital cascade auditory filter model proposed by Richard Lyon and described in detail in (Lyon, 2017). It closely approximates the physiological elements that consist of the human cochlea and mimics its qualitative behavior. The CAR part models the BM function that translates the cochlear fluid pressure wave (converted from the sound wave by the middle ear) into positions of maximal displacement along its length. Its pole-zero cascade form uses fewer parameters in the z domain than other filters, such as the gammatone and the gammachrip filters (impulse response) to provide an excellent fit to data on human detection tones in masking noise (Lyon, 2011). The FAC part models the OHC, the IHC and the medial olivocochlear efferent system functions that transduce the cochlear mechanic vibrations into electronic signals and exert a nonlinear gain control feedback on the BM through the OHC. The FAC nonlinear effects include a fast wide-dynamic-range compression and frequency distortions such as cubic difference tones (CDTs) and quadratic difference tones (QDTs) and are realized by moving the positions of the poles and zeros of the CAR resonators in the z plane.

Saremi et al. compared seven computational cochlear models including one cascade filterbank model (CAR-FAC), one transmission-line model, one biophysical model, and four parallel filterbank models (Saremi et al., 2016) in response to a set of common stimuli, which are used in the clinical assessment of human hearing to study their performance. The results show that the CAR-FAC exhibits an outstanding agreement with the biological data recordings at a reasonably low computational cost. These factors formed our basis of developing the CAR-FAC model and investigating its characteristics and possible applications.

We target a digital ASIC implementation of the CAR-FAC model for machine hearing applications since it is small, more energy efficient and more stable than analog implementations (Sarpeshkar, 2006). For the validation and prototype stage, we choose to implement it on a small FPGA board, the Altera Cyclone V starter kit. We previously introduced the CAR-FAC system on FPGA in (Xu et al., 2016), and here we present the complete system and measurement results.

## Materials and methods

### The CAR-FAC model

**Figure 2:**
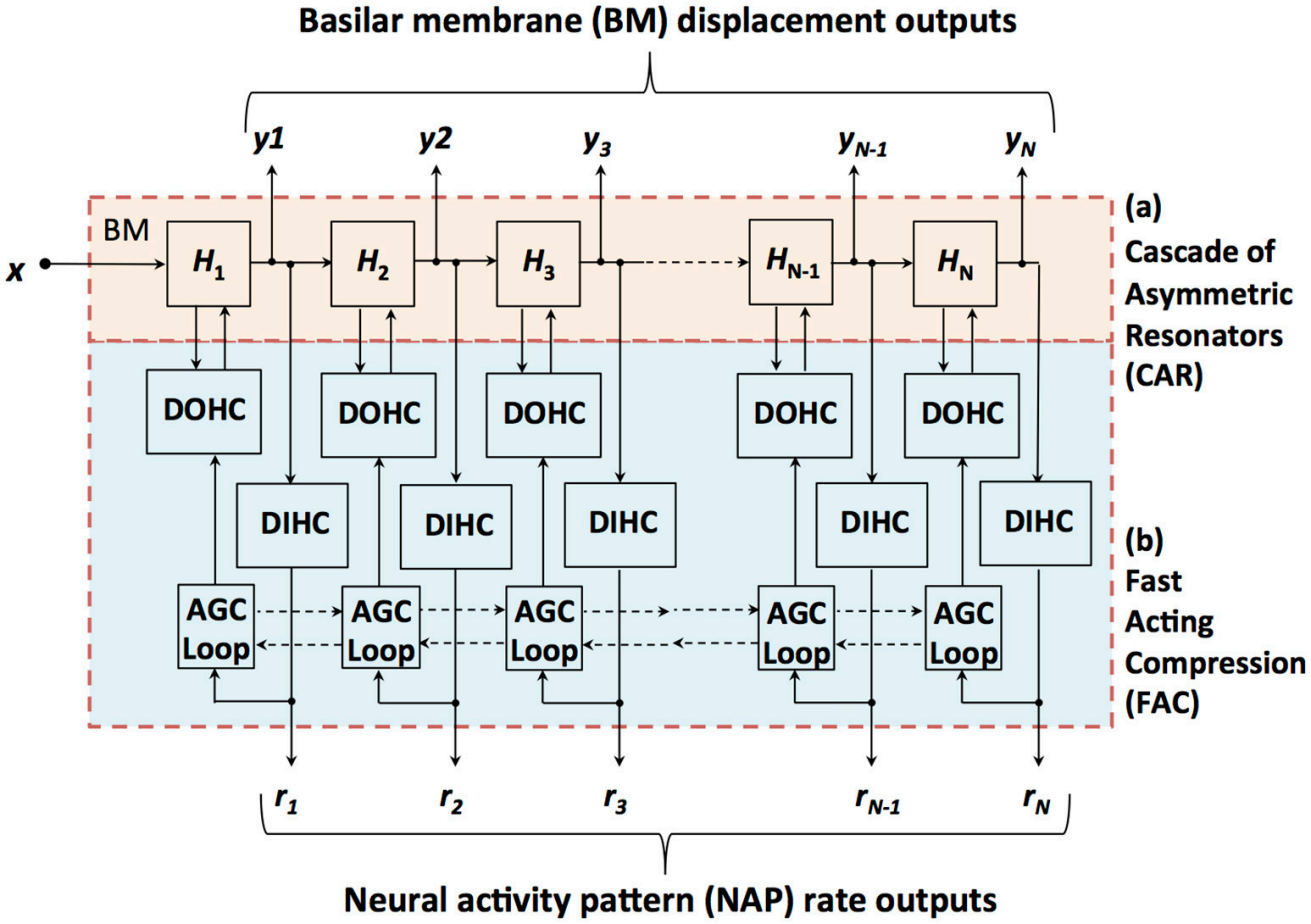
Structure of the CAR-FAC model. *x* is the input sound, *H*_1_ to *H*_*N*_ are the transfer functions of the CAR part, and *y*_1_ to *y*_*N*_ represent the CAR-FAC output. The CFs of the CAR resonators decrease from left to right. The DOHC, the DIHC and the AGC loop comprise the FAC part. The neural activity pattern (NAP) rate outputs, *r*_1_ to *r*_*N*_, are estimations of average instantaneous nerve firing rates.

The CAR-FAC model consists of a cascade of asymmetric resonators, a digital OHC (DOHC) model, a digital IHC (DIHC) model and an AGC loop, as shown in Figure 2. At each stage, the resonator *H*_*i*_ is connected to its next stage and the DIHC. It also gives an intermediate variable, velocity, to the DOHC. The DIHC feeds back to the DOHC through the AGC loop. The DOHC combines the AGC loop output and the velocity and feeds back to the resonator. The CAR-FAC output includes a multi-channel BM out *y*_*i*_ and a DIHC out, which can be transformed into the neural activity patterns *r*_*i*_. The details of each model are described hereafter:

#### CAR

**Figure 3:**
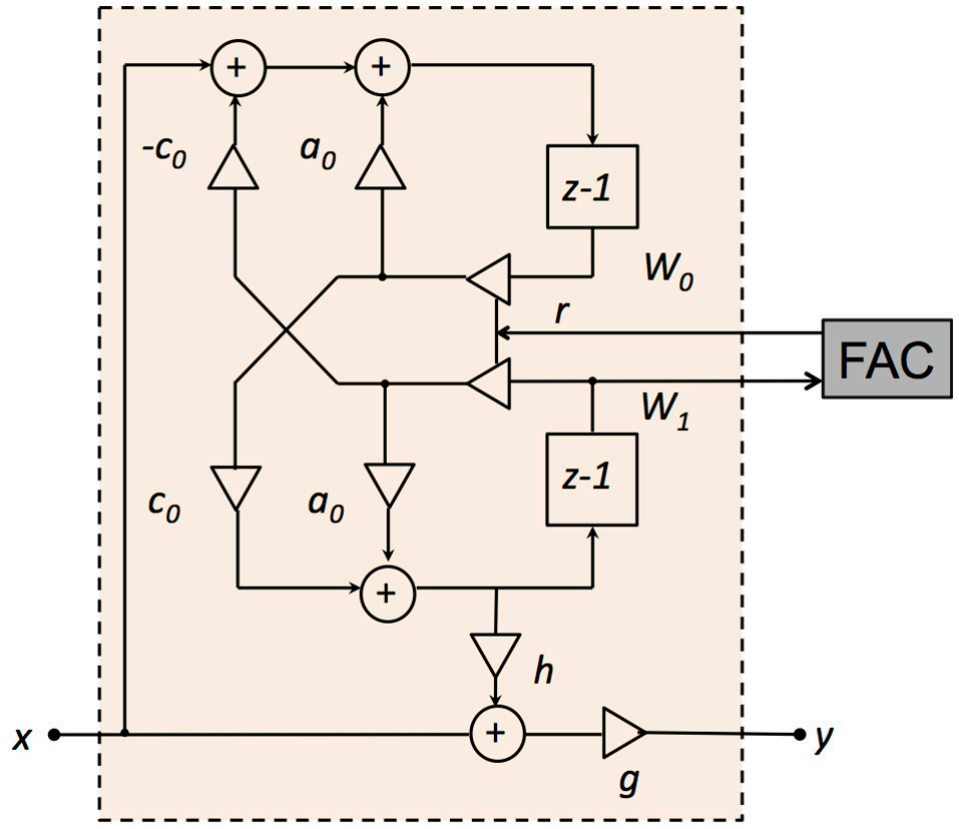
Structure of the two-pole-two-zero resonator. *a*_0_, *c*_0_, and *h* are the resonator coefficients, *r* is the pole/zero radius in the z plane, *g* is the DC gain factor, *W*_0_ and *W*_1_ are the intermediate variables, *x* is the input, and *y* is the output.

In the CAR, the asymmetric resonator is a coupled form two-pole-two-zero filter, as shown in Figure 3. The transfer function of the filter in the z domain is:

(1) $$\begin{array}{lll} H\left(z\right) & = & \frac{Y}{X}=g\left[\frac{\left(z-z_{zero}\right)\left(z-z_{zero}^{*}\right)}{\left(z-z_{pole}\right)\left(z-z_{pole}^{*}\right)}\right]\\ = & g\left[\frac{z^{2}+\left(-2a_{0}+hc_{0}\right)rz+\;r^{2}}{z^{2}-2a_{0}rz+\;r^{2}}\right] \end{array}$$

The two-pole coupled form has a pair of conjugate poles ($z_{pole}\;and\;{\;z}_{pole}^{*}$):

(2) $$\begin{array}{lll} z_{pole},z_{pole}^{*} & = & \frac{2a_{0}r\pm \sqrt{{\left(2a_{0}r\right)}^{2}-4r^{2}}}{2}\\ = & rcos\left(\theta_{R}\right)\pm irsin\left(\theta_{R}\right) \end{array}$$

(3) $$\begin{array}{l} a_{0}=\;\cos\left(\theta_{R}\right) \end{array}$$

where θ_*R*_ is the pole angle in the z plane. The conjugate zeros (*z*_*zero*_ and $z_{zero}^{*}$) are:

(4) $$\begin{array}{lll} z_{zero},\;z_{zero}^{*} & = & \frac{-\left(-2a_{0}+hc_{0}\right)r\pm \sqrt{{\left(\left(-2a_{0}+hc_{0}\right)r\right)}^{2}-4r^{2}}}{2}\\ =rcos\left(\theta_{z}\right)\pm irsin\left(\theta_{z}\right) \end{array}$$

(5) $$\begin{array}{l} a_{0}-hc_{0}/2=\;\cos\left(\theta_{Z}\right) \end{array}$$

where θ_*Z*_ is the zero angle in the z plane. The zero radius is the same as the pole radius, *r*. The condition for complex zeros becomes relevant for high-frequency channels, where cos(θ_*R*_) < 0:

(6) $$\begin{array}{lll} a_{0} & - & \frac{hc_{0}}{2}>\;-1 \end{array}$$

(7) $$\begin{array}{lll} h & < & \frac{2+2a_{0}}{c_{0}} \end{array}$$

Coefficient *g* controls the stage DC gain. Here, *g* is set to maintain a unit DC gain for each stage of the filterbank:

(8) $$\begin{array}{l} g=\;\frac{1}{H\left(1\right)}=\;\frac{1-2a_{0}r+r^{2}}{1-2\left(a_{0}-hc_{0}\right)r+r^{2}} \end{array}$$

In this structure, the zeros can be moved together with the poles by changing *r* while keeping *h* constant. The two zeros are placed slightly above the poles in frequency, and the distance between the zeros and the poles are set by the coefficient *h*. For lower *h*, the zeros are close to the poles, forming a steeper roll-off (asymmetric). For higher *h*, the zeros are further away from the poles, which results in a gradual roll-off at the higher frequency end. The steeper roll-off fits the auditory filtering characteristic and provides better frequency selectivity. Here, *h* is set to *c*_0_ to keep the zero frequency at half an octave above the pole frequency.

Additionally, changing the poles and the zeros of the filter, via *r* leaves the zero-crossing times of the filter's impulse response nearly unchanged in time. The unchanged zero crossing characteristic satisfies the physiologically observed condition that the impulse response zero crossings are very nearly unchanged with variation in stimulus level (Lyon, 2017).

The zeros and poles are set initially for each cascade stage. The poles of the two-pole-two-zero resonator are chosen to be equally spaced along the normalized length of the cochlea according to the Greenwood map function (Greenwood, 1990):

(9) $$\begin{array}{l} f=165.4\left(10^{2.1x}-1\right) \end{array}$$

Here, coefficient *x* is the normalized position along the cochlea, varying from 0 at the apex of the BM, to 1 at the basal end, and coefficient *f* is the pole frequency.

In the CAR-FAC model, the FAC effects are achieved by moving the initial CAR poles and zeros positions by varying their radius *r*. The details of each element in the FAC part are presented in the next three sections.

#### DOHC

**Figure 4:**
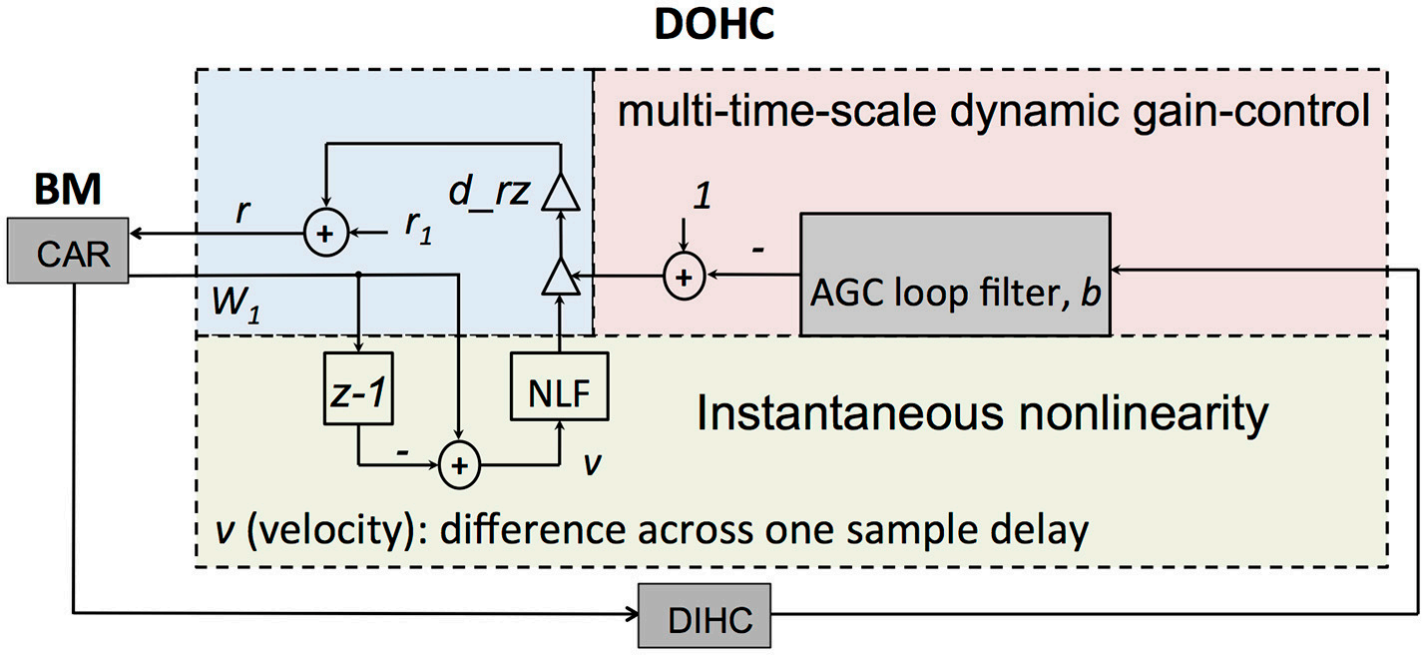
Structure of the DOHC model. The instantaneous nonlinearity performs a nonlinear gain control (NLF) on the CAR velocity, which is calculated from the BM coefficient *W*_1_. The multi-time-multi-scale dynamic gain-control factor, *b*, is obtained from the AGC loop. Both gain control factors are combined to change *r* through Equation (10).

The DOHC models the OHCs function, actively and nonlinearly amplifying the wave propagation in the cochlea. In the CAR-FAC model, the DOHC gain control mechanism integrates a local instantaneous nonlinearity and a multi-time-scale nonlinearity, as shown in Figure 4. The instantaneous nonlinearity is based on the BM velocity, taken as the rate of change of *W*_1_. The multi-time-scale nonlinearity comes from the DIHC feedback through the AGC loop filter. Both combine to change the pole (zero) radius *r*:

(10) $$\begin{array}{l} r=r_{1}+d_{rz}\times \left(1-b\right)\times NLF\left(v\right) \end{array}$$

where coefficient *r*_1_ is the minimum radius, corresponding to the maximumdamping of the resonator. In a digital implementation, *r*_1_ is given by:

(11) $$\begin{array}{l} r_{1}=1-damping\times \left(\frac{2\pi f}{f_{s}}\right) \end{array}$$

where the coefficient *damping* controls the damping factor, *f* is the CF from Equation (9), and *f*_*s*_ is the sampling frequency. *r*_1_ keeps the damping away from zero, thereby keeping the system away from the Hopf bifurcation of the resonators. *r*_1_ also makes the damping bounded. The increment of *r* above *r*_1_ is the relative undamping. It is the product of the nonlinear function (*NLF*) of the CAR velocity, and the AGC loop, *b*. The coefficient *d*_*rz* controls the rate at which the velocity and the AGC loop affects the damping. Here, *d*_*rz* is set to 0.7 × (1-*r*_1_) (Lyon, 2017).

The *NLF* function in the DOHC is given by:

(12) $$\begin{array}{l} NLF\left(\nu \right)=\;\frac{1}{1+{\left(\nu \times scale+offset\right)}^{2}} \end{array}$$

where ν is the CAR velocity, *scale* is 0.1, and *offset* is 0.04 (Lyon, 2017). At high velocities, the velocity-squared function grows very rapidly and saturates the *NLF* toward zero, thus making the damping saturate toward a high-level limit.

The level dependence of the damping mechanism introduces frequency distortions. The velocity-squared function includes a double-frequency term that interacts with the CAR coefficients (*a*_0_*r* and *c*_0_*r*) to generate a CDT. For example, if there are two tones, *f*_1_ and *f*_2_ (where *f*_1_ < *f*_2_), then a third tone, at the frequency (2*f*_1_–*f*_2_) will appear and propagate through the cascade of filters. The *offset* in the NLF function introduces a first order damping factor, which will interact with the CAR coefficients to generate a QDT, (*f*_2_–*f*_1_) (Lyon, 2017).

#### DIHC

**Figure 5:**
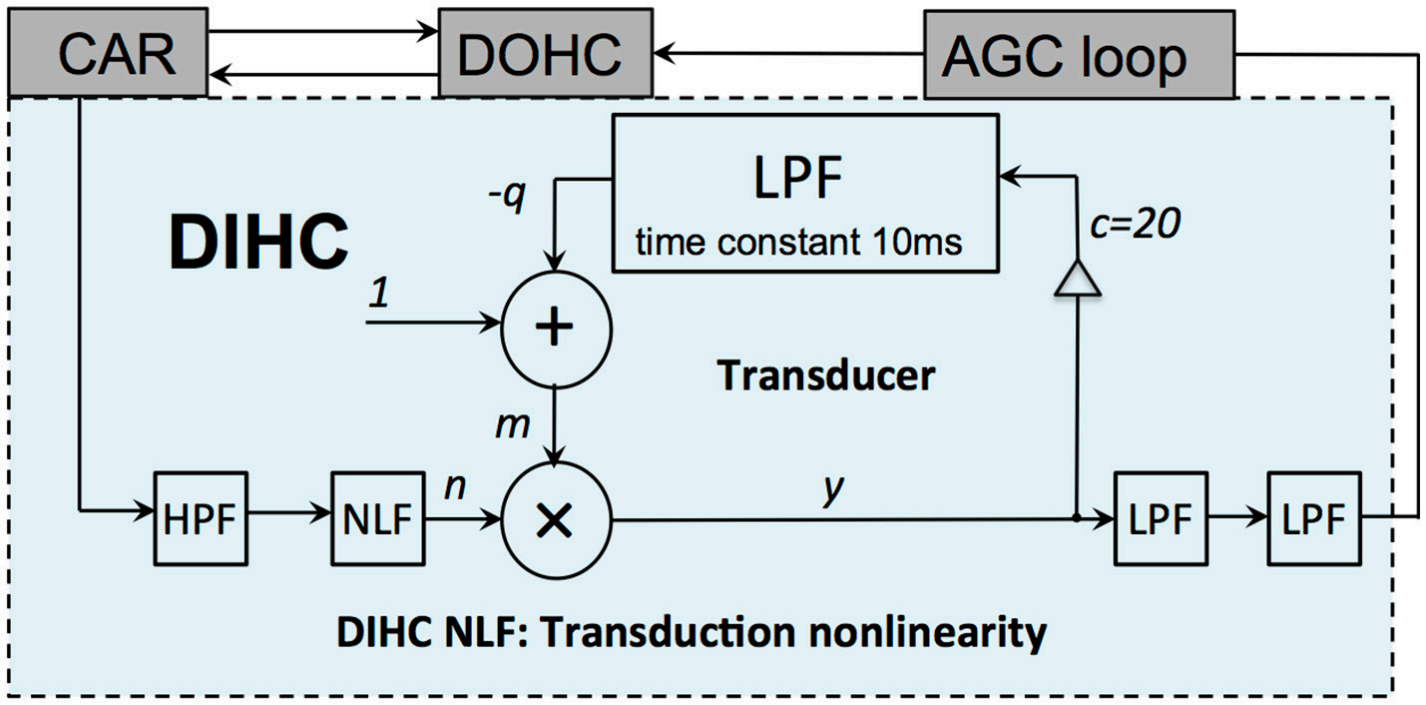
Structure of the DIHC model. It comprises a HPF, a transduction nonlinearity unit, a transducer unit and two LPFs.

The DIHC models the IHC function. It comprises a high-pass filter (HPF), a transduction nonlinearity unit, a transducer unit and two LPFs. The IHCs are mechano-electrical transducers that sense the BM vibration, convert the mechanical motion into electrical signals, and deliver the results to the nervous system. The DIHC model is shown in Figure 5. The HPF suppresses the CAR output frequencies below 20 Hz. The transduction nonlinearity includes a half wave rectifier (HWR), and a rational sigmoid function:

(13) $$\begin{array}{lll} u & = & HWR\left(BM_{hpf}+0.175\right) \end{array}$$

(14) $$\begin{array}{lll} n & = & \frac{u^{3}}{u^{3}+\;u^{2}+0.1} \end{array}$$

where *BM*_*hpf*_ is the high pass filtered CAR output, *u* is the intermediate variable, and *n* is the transduction nonlinearity output. The HWR mimics directional sensitivity of the IHC transduction which response mainly in one direction. The constant 0.175 (Lyon, 2017) keeps the nonlinearity at a fixed value at zero response. The rational sigmoid function (14) provides a nearly linear response at low amplitudes and a saturating response at higher amplitudes.

The transducer unit detects and amplifies the signal onset, then compresses and reduces its response gain quickly after the signal onset. It is implemented by:

(15) $$\begin{array}{lll} m & = & 1-q \end{array}$$

(16) $$\begin{array}{lll} y & = & nm \end{array}$$

(17) $$\begin{array}{lll} q_{new} & = & \left(1-a\right)q+a\left(cy\right) \end{array}$$

where *m* is the adaptive gain of its input, *n, c* is set to 20, and *q* is the LPF state. The time constant of the first order FIR LPF is set to 10 ms. The final two FIR LPFs smooth the output using a time constant of 80 μs each.

#### AGC loop

**Figure 6:**
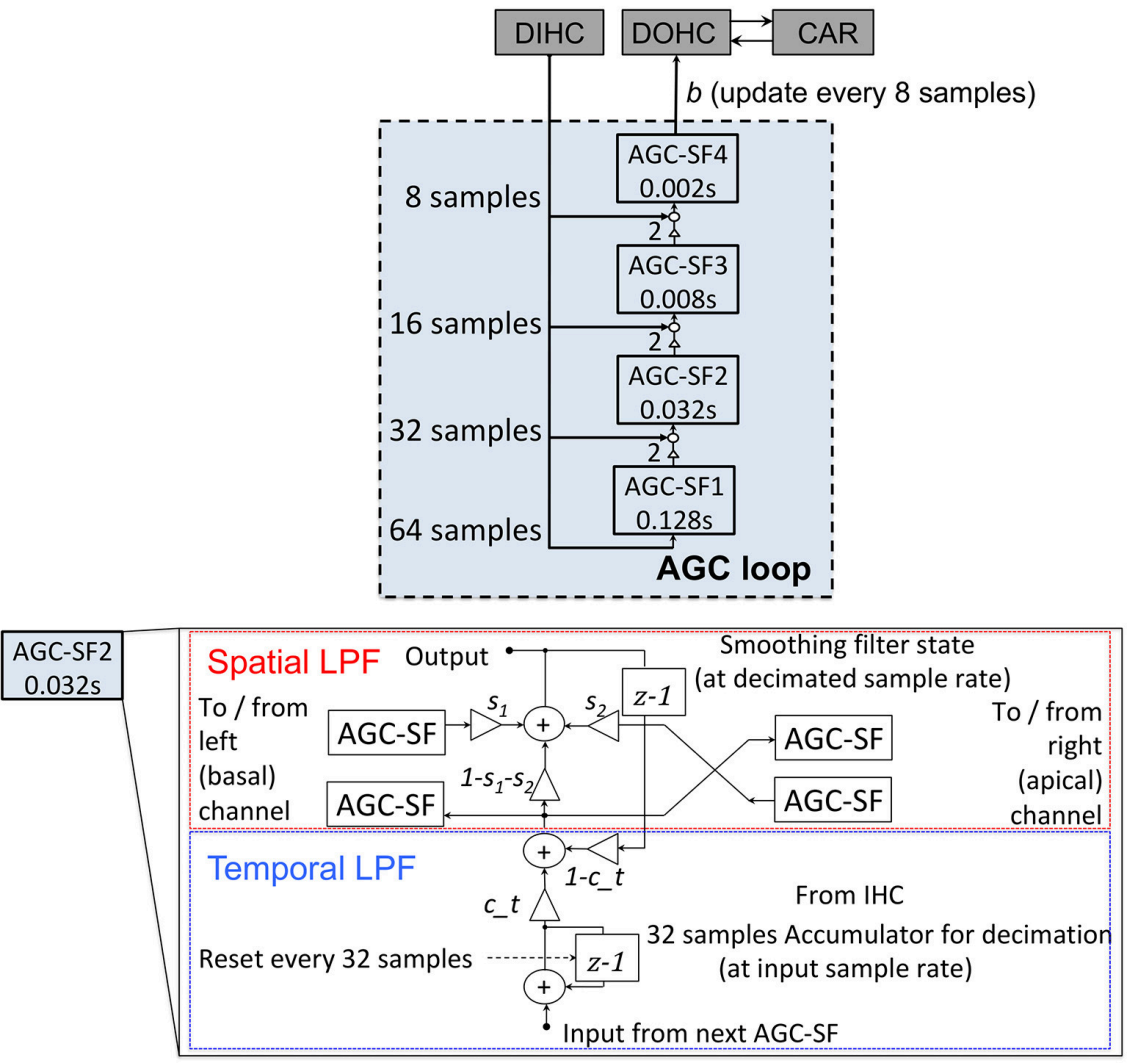
Structure of the AGC loop. Four stages of the temporal smoothing filters (SF) (Upper). Each stage consists of a temporal LPF with a defined time constant (0.002, 0.008, 0.032, and 0.128 s) and a three-tap spatial smoothing filter. The internal structure of an AGC-SF (Lower), the input of the AGC-SF comes from the lower filter stage with the smaller time constant as well as the accumulation of the DIHC. The output goes to the next stage of the temporal filter. The spatial smoothing filter is a three-tap smoothing filter coupled with lateral channels. *s*_1_, *s*_2_, and *1*-*s*_1_-*s*_2_ are the spatial filter coefficients. *c_t* is the temporal LPF coefficient calculated from the time constant.

The AGC loop consists of a four-stage cascade FIR LPF, with each stage coupled with its left and right neighbors to form a three-stage spatial LPF. It feeds the DIHC signal back to the DOHC at a much lower update rate than other parts of the CAR-FAC model. The AGC loop models the medial olivocochlear system's efferent feedback that exerts an AGC on the BM vibration through the OHCs. The AGC loop filter is shown in Figure 6. Each AGC smoothing filter (SF) stage includes a temporal linear LPF with a defined coefficient *c_t* and a three-tap spatial LPF. The three-tap spatial LPF coefficients [*s*_1_, *1*-*s*_1_-*s*_2_, *s*_2_] apply weight *s*_1_ to the left neighbor value, *s*_2_ to the right neighbor value, and *1*-*s*_1_-*s*_2_ to the current channel value to keep the total mixing gain equal to 1. For a 44.1 kHz signal, in the fastest and most local stage, AGC-SF4, *c_t* is set to 0.09, *s*_1_ is 0.14 and *s*_2_ is 0.2 (Lyon, 2017). The input of each AGC-SF comes from a respective accumulation of the DIHC and its lower stage. The AGC-SF4 output *b* feeds back to the DOHC.

### FPGA implementation

**Figure 7:**
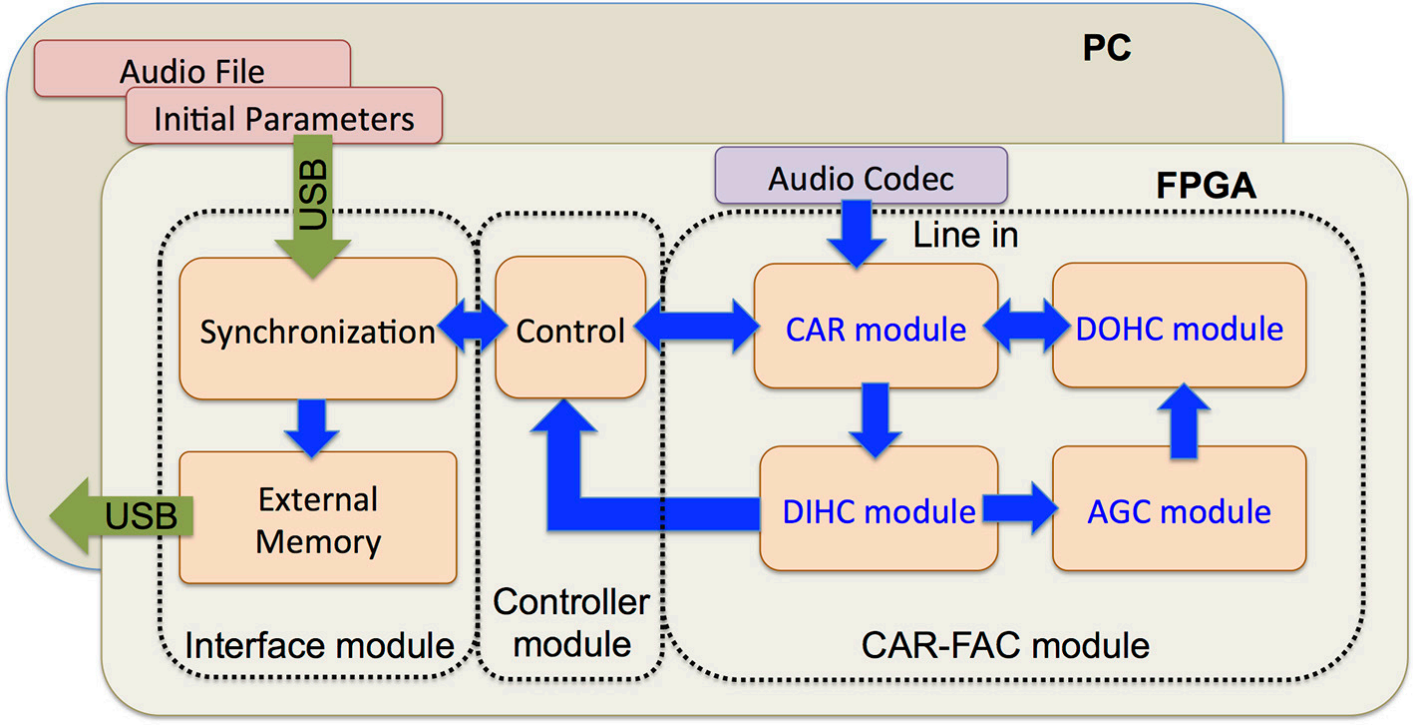
Architecture of the CAR-FAC FPGA system. The system consists of an audio codec, a CAR-FAC module, a controller module and an interface module. The FPGA board is hosted by a PC through a USB interface.

The CAR-FAC system can be efficiently implemented on FPGA, and the system is configurable in filter parameters and channel numbers Figure 7 shows the architecture of the system. It comprises an audio codec, a CAR-FAC module, a controller module and an interface module. The system provides two ways of sound input. One way is through the SSM2603 audio codec on the FPGA board. It also supports recorded audio file input from the PC host through a USB 3.0 interface.

The CAR-FAC module implements the components described in section The CAR-FAC Model. Additionally, the CAR module can operate independently: when the FAC function is turned off, the DOHC and AGC loop function will be switched off, and all the CAR coefficients (*a*_0_, *c*_0_, *g, h*, and *r*) remain fixed at their initial values. The system then operates as a linear CAR system.

The controller module controls the system data flow, including writing the initial coefficients, and/or the audio file input to the CAR-FAC module, as well as the CAR-FAC module output to the interface module. Additionally, the output of the system is selectable: we can choose either the BM output or the DIHC output as the system output.

The interface module consists of a data synchronization module, an external memory, and a USB interface. The data synchronization circuit synchronizes data between different clock domains. There exist two clock domains in the system: a system clock domain (250 MHz) and an interface clock domain (100 MHz). The system clock domain includes the controller module and the CAR-FAC module. The interface clock domain is unique to the interface module. The external memory is a 1 GB DDR3 SDRAM on the FPGA board: it stores the CAR-FAC output data. The USB interface communicates between the FPGA board and the PC, and transmits the system's initial coefficients (*a*_0_, *c*_0_, *g, h, r, r*_1_, *b*, and *d_rz*), and, if required, the input audio file from the PC to the FPGA board. It also transmits the system's output from the external memory to the PC.

**Figure 8:**
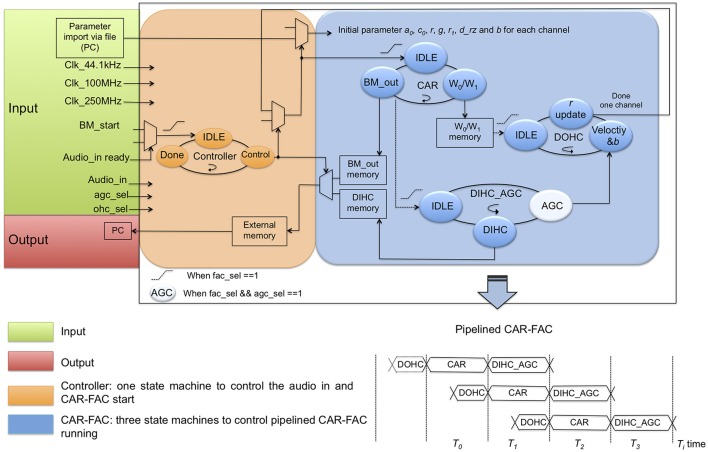
CAR-FAC system design diagram. The CAR-FAC system is implemented with 20-bit word length for the design coefficients, BM output, and DOHC output, and 14-bit for the DIHC output and the AGC output. The controller state machine determines the cochlear channel to be processed at any particular time and controls the CAR-FAC coefficients and data for that channel. The BM_start signal controls the start of the system through the controller, and it is triggered by the Audio_in_ready signal. The ohc_sel is a selector switch for the CAR/CAR-FAC function. The agc_sel is a switch for the AGC loop function. The CAR state machine calculates the transfer function of Equation (1) and controls the DOHC and DIHC_AGC start in the system. The DOHC state machine calculates Equation (10–12) and feeds back an updated *r* to the CAR. The DIHC-AGC calculates Equation (13–17), as well as the AGC_loop function shown in Figure 6. The AGC output *b* feeds back to the DOHC module via Equation (10). The pipelined CAR-FAC timing diagram is shown in lower right.

We first simulated the CAR-FAC model in Python with floating-point numbers. Next, we verified the model using the fixed-point numbers to determine the required word length for the FPGA implementation. We use 20-bit BM variables, 20-bit DOHC variables, 14-bit DIHC variables and 14-bit AGC variables to approximate the floating-point CAR-FAC performance and to meet the input, output and internal variables range to achieve a 70 dB input dynamic range. We use the pipeline technique to parallel the CAR module, the DOHC module, and the DIHC_AGC module, and the time-multiplexing approach to reuse single CAR, DOHC, and DIHC_AGC hardware module to implement a compact reconfigurable CAR-FAC system. The system design diagram is shown in Figure 8.

In digital audio, 44.1 kHz is a common sampling frequency, and the digital hardware of the CAR module (the two-zero-two-pole resonator) and the FAC module (the DOHC module and the DIHC-AGC module) can operate much faster than the audio sample interval (22.68 μs). Hence, in this system, a single CAR-FAC hardware module is reused multiple times to implement the multiple-channel multi-level pipeline CAR-FAC system. At 44.1 kHz sampling frequency, with a single CAR-FAC module, we were able to implement up to 70 filter channels real-time CAR-FAC system.

For each CAR-FAC module, there exist four state machines in the system. The controller state machine determines the cochlear channel to be processed at a particular time and controls the CAR-FAC coefficients and data for that channel. The CAR state machine calculates the transfer function of Equation (1). The DOHC state machine calculates Equation (10–12), and feeds back an updated *r* to the CAR. The DIHC-AGC state machine calculates Equation (13–17), as well as the AGC_loop function shown in Figure 6. The AGC output *b* feeds back to the DOHC module via Equation (10).

The BM_start signal controls the start of the system through the controller and is triggered by the Audio_in_ready signal. If there exists an audio input (Audio_in) from either the PC or the audio codec, the BM_start signal will be sent to the CAR through the controller, and the CAR will start to run. The ohc_sel is a selector switch for the CAR/CAR-FAC function, and the agc_sel is a switch for the AGC loop function. When the ohc_sel is low, the DOHC function is switched off, and the CAR-FAC operates as a linear CAR system, and we can choose either the CAR or the DIHC as the output. When both the ohc_sel and the agc_sel are high, the whole CAR-FAC function is switched on. When the ohc_sel is high and the agc_sel is low, the AGC loop function is switched off, leaving only the instantaneous nonlinearity in the CAR-FAC system.

The CAR state machine controls the DOHC and DIHC_AGC start in the system. It will send a start signal to the DOHC and the DIHC-AGC module separately at a particular time to start the DOHC and the DIHC-AGC function if both the ohc_sel and the agc_sel are high. The DOHC state machine starts when the CAR module finishes updating the internal variables *W*_0_/*W*_1_. The DIHC-AGC state machine starts when the BM output calculation is finished. The pipelined CAR, DOHC, and DIHC_AGC structure is shown in Figure 8 bottom right. Each filter channel output, BM_out or DIHC_out, is moved to the external memory in the interface module and sent to the PC through the USB interface.

The device utilization for a single CAR-FAC module is shown in Table 1. Given the size of a Cyclone V FPGA and the low hardware resource utilization of a single CAR-FAC hardware module, this FPGA board can accommodate up to a total of 210 cochlear channels (using three CAR-FAC hardware modules).

**Table 1:** Device utilization summary.

	**Used**	**Available**	**Utilization (%)**
ALM	5,235	29,080	18
Memory (bits)	1,082,812	4,567,040	24
DSPs	49	150	33

## Results

### CAR-FAC transfer function

We have implemented a real-time digital CAR-FAC system at a 44.1 kHz sampling rate on a Cyclone V FPGA board covering an input frequency range up to 22.05 kHz. The number of channels in the system is reconfigurable, and more channels will result in more overlap among filters if the frequency range is kept the same. For machine hearing applications, about 50% overlap in items of equivalent rectangular bandwidth (ERB) is considered to provide a well-behaved representation of a sound (Lyon, 2011). Psychophysical experiments (Glasberg and Moore, 1990; Moore, 1995) show that each ERB at moderate sound level corresponds to about 0.89 mm on the BM. Therefore, for the total length of the human BM (about 35 mm), this would correspond to 78 channels with 50% overlap, or 11 channels per octave according to the Greenwood function map in Equation (9). Machine hearing models typically use 60 to 100 channels in total (Lyon, 2011), here we implemented a 70-channel CAR-FAC system and investigated the system characteristics.

**Figure 9:**
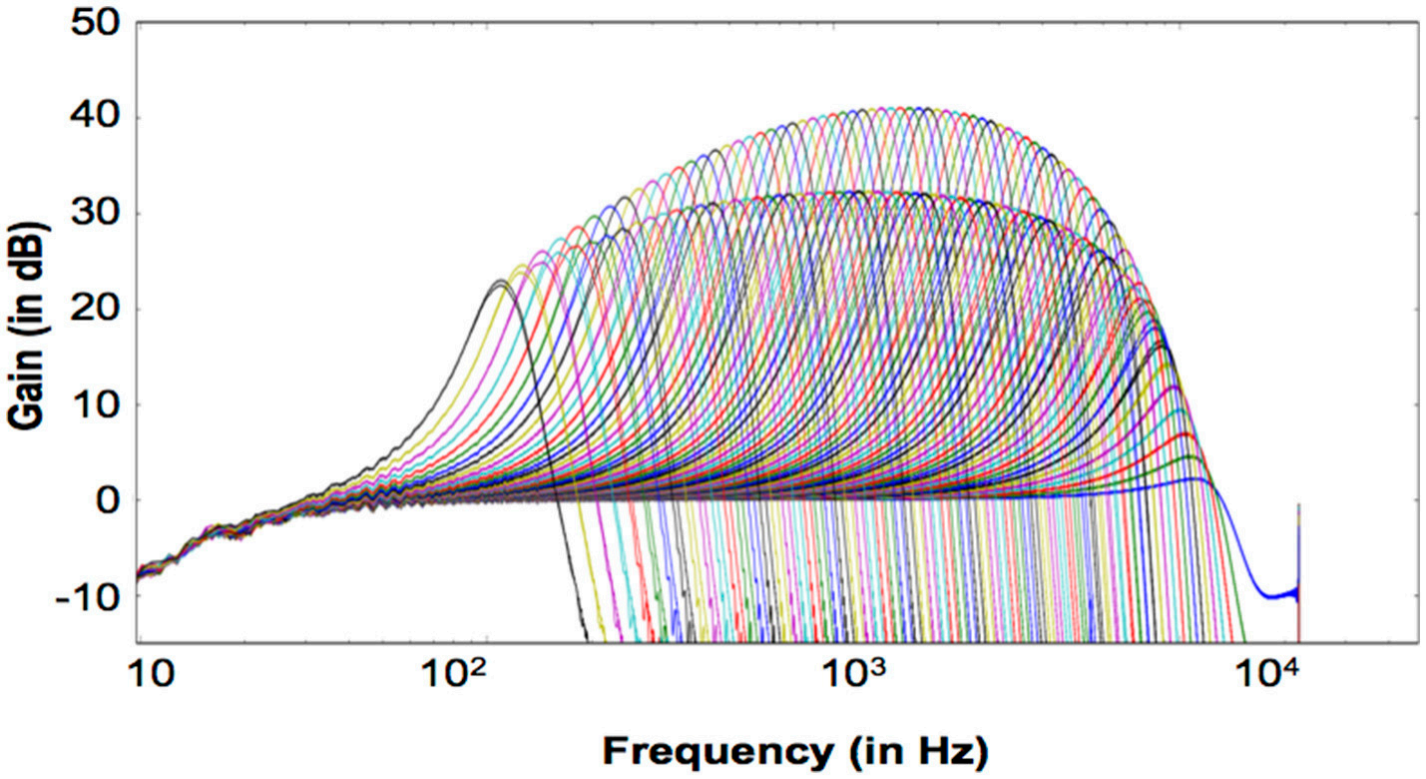
Transfer function of the 70-channel CAR-FAC system to a -40 dB FS, 1 s sine tone sweep from 20 Hz to 22.05 kHz (squared-cosine rise and decay time of 0.1 s to minimize the influence of the spectral splatter). The CAR response (Upper) when the FAC function is switched off; The CAR-FAC response (Lower).

The measured system transfer function in response to a -40 dB full scale (FS), 1 s sine tone sweep from 20 Hz to 22.05 kHz (squared-cosine rise and decay time of 0.1 s to minimize the influence of the spectral splatter) is shown in Figure 9. Note that we express the intensity of input signals in dB FS relative to a maximum amplitude of FFFFF (20-bit unsigned number), and the input amplitude is normalized to 1.0 in the figures in this paper. The upper set of curves shows the linear CAR response of all the 70 channels when the FAC function is switched off. The lower set shows the CAR-FAC response. Both the CAR and the CAR-FAC show an increased gain in the lower and moderate frequency range and a reduced gain in the higher frequency range. Additionally, the FAC function shows a global gain compression effect on the system response.

**Figure 10:**
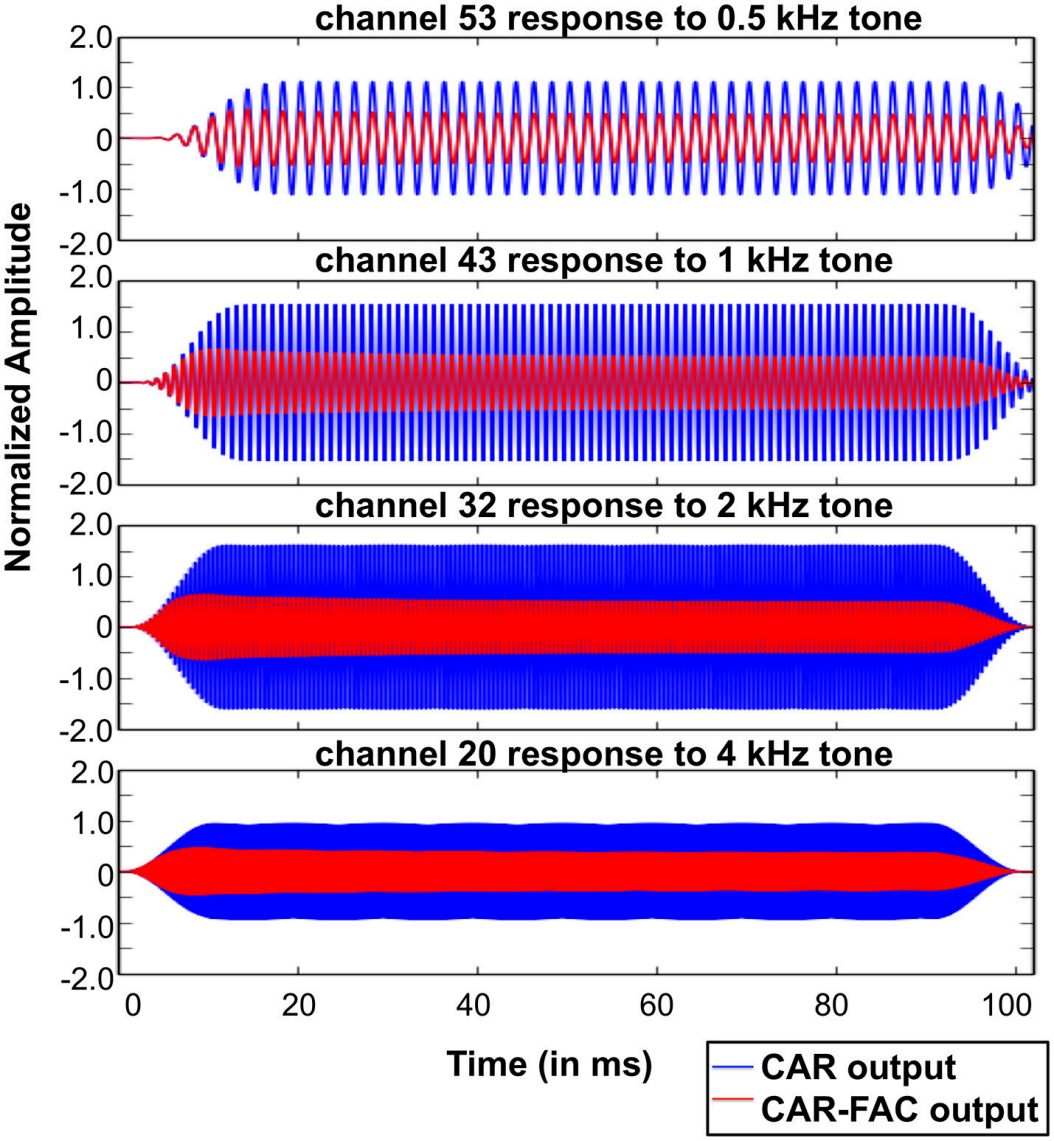
CAR and CAR-FAC output in response to 0.5, 1, 2, and 4 kHz tones with an amplitude of -40 dB FS at the channels of CFs corresponding to the input frequencies.

Figure 10 shows the CAR and the CAR-FAC output in the time domain in response to 0.5, 1, 2, and 4 kHz tones (squared-cosine rise and decay time of 10 ms) at channels of CFs corresponding to the input tones. The CAR amplifies the amplitude of the input tones linearly, whereas the CAR-FAC responses exhibit a gradually compressed gain control.

### CAR-FAC excitation patterns and nonlinear growth

Excitation patterns show the vibration amplitude across the BM to a single sound. Here, the excitation patterns were calculated as the root-mean-square (RMS) signal at the output of all the CAR-FAC channels (Ren, 2002). The Greenwood function in Equation (9) was used as the position-frequency map.

**Figure 11:**
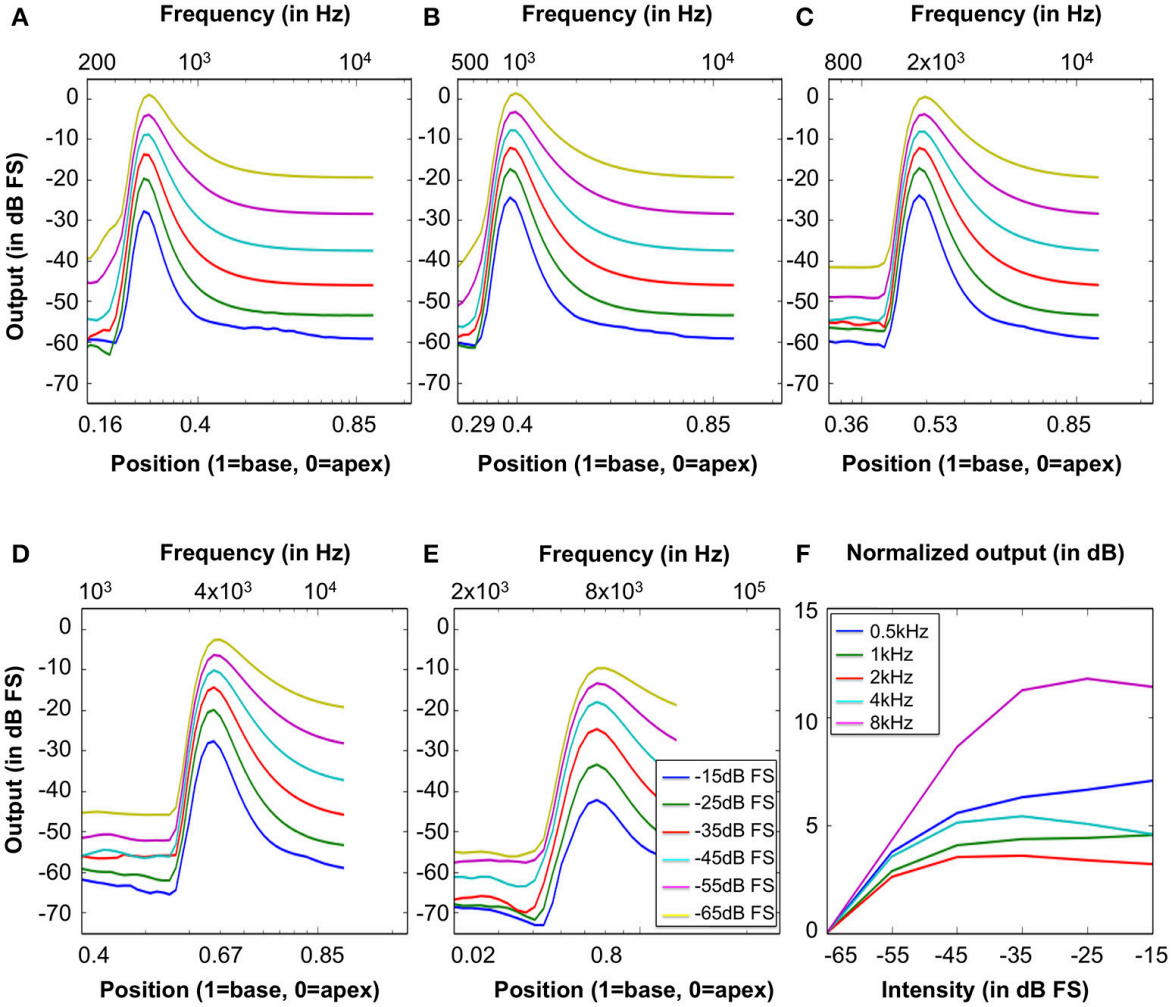
Excitation patterns calculated as the RMS output signal of the 70 CAR-FAC channels in response to tones at **(A)** 0.5 kHz, **(B)** 1 kHz, **(C)** 2 kHz, **(D)** 4 kHz, and **(E)** 8 kHz with intensities ranging from -65 dB FS to -15 dB FS in steps of 10 dB FS. The x-axis shows both the frequency and the position-frequency location calculated from Equation (9). **(F)** The normalized nonlinear response growth of the system to the tones of 0.5, 1, 2, 4, and 8 kHz (squared-cosine rise and decay time of 10 ms) with intensities between -65 dB FS and -15 dB FS in steps of 10 dB FS.

Figures 11A–E show excitation patterns in response to 100 ms tones at 0.5, 1, 2, 4, and 8 kHz (squared-cosine rise and decay time of 10 ms) with intensities ranging from -65 dB FS to -15 dB FS in steps of 10 dB FS. The peak locations of all excitation patterns correspond to the input tones through the position-frequency map, demonstrating that the system captures the human frequency-position map well.

Additionally, we calculated the BM input/output (I/O) function to evaluate the nonlinear and compression effects of the system. The I/O function is the ratio between the RMS output at the CF channel corresponding to the stimulus frequency and the RMS of the stimulus (Saremi et al., 2016). Figure 11F shows the I/O function curves of the system to 100 ms pure tones of 0.5, 1, 2, 4 and 8 kHz (squared-cosine rise and decay time of 10 ms) with intensities between -65 dB FS to -15 dB FS in steps of 10 dB FS. The I/O curves were normalized with respect to the -65 dB FS I/O point. The output shows a compressed intensity range (15 dB FS) comparing to the input (50 dB FS), and the I/O curves were generally more compressive at moderate CFs, such as 1, 2, and 4 kHz, than the lower and higher CFs (0.5 and 8 kHz).

### CAR-FAC frequency selectivity and *Q* tuning

The CAR-FAC frequency selectivity was evaluated from the system frequency responses. The frequency response was calculated using the FFT from the system impulse responses at the channels of CFs corresponding to 0.5, 1, 2, 4, and 8 kHz.

Furthermore, in the CAR-FAC system, quality factor (*Q* factor) tuning is achieved by tuning of the damping factor [*damping* in Equation (11)]. Here, to investigate the system's *Q* tuning effects, we used different damping factors and calculated the corresponding *Q* factors associated with the ERB, *Q*_*ERB*_ (de Boer and Nuttall, 2000):

(18) $$\begin{array}{l} Q_{ERB}=\frac{CF}{ERB} \end{array}$$

The ERB was evaluated from the system's impulse response power spectral density (PSD).

**Figure 12:**
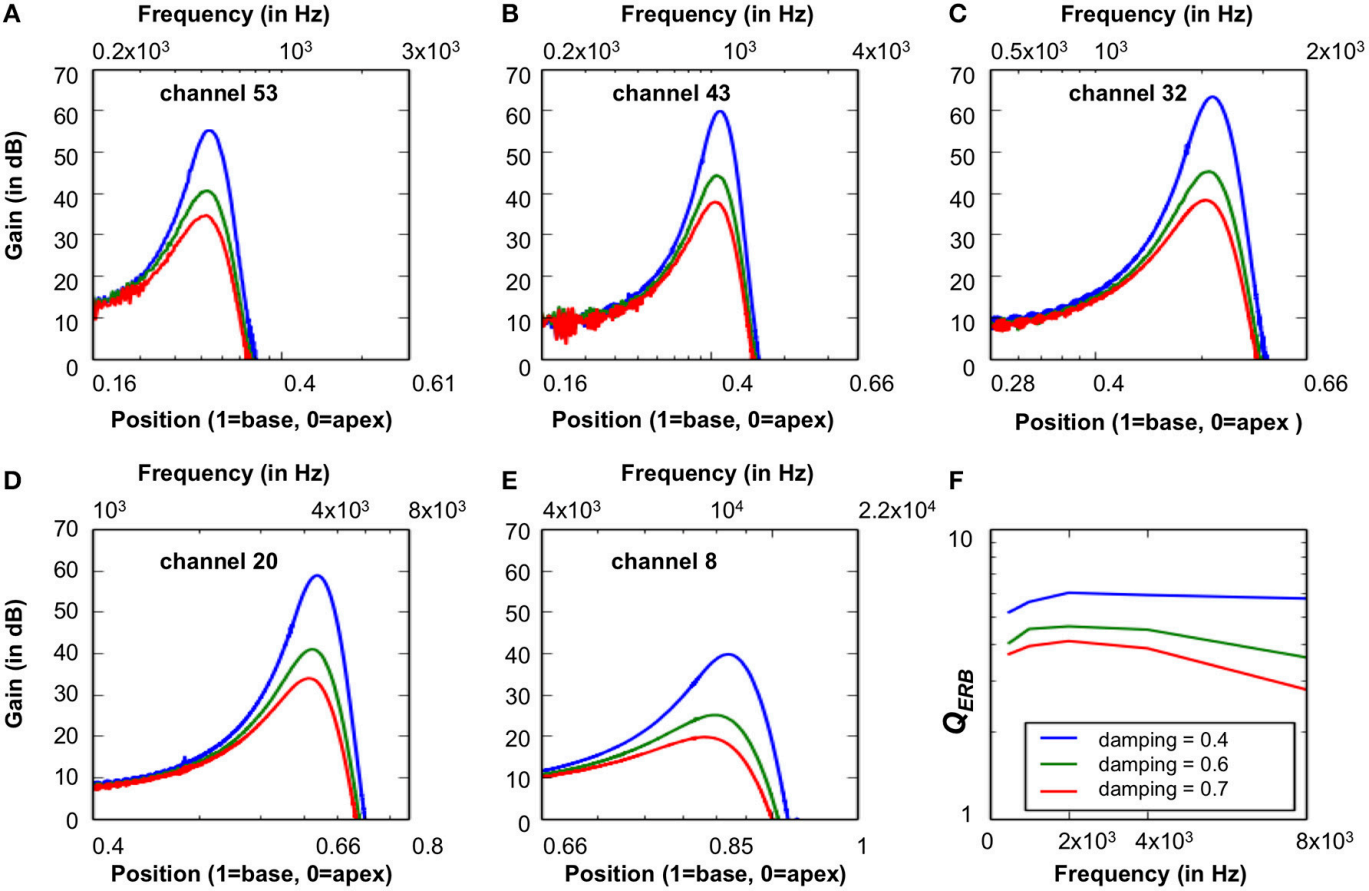
**(A–E)** The CAR-FAC system response calculated at the CFs corresponding to 0.5, 1, 2, 4, and 8 kHz with three damping factors (0.4, 0.5, and 0.7) in Equation (11). The x-axis shows both the frequency and the BM location calculated from Equation (9). **(F)** The corresponding *Q*_*ERB*_ at CFs corresponding to 1, 0.5, 2, 4, and 8 kHz estimated from the BM impulse response PSD at CFs.

Figures 12A–E shows the system's frequency responses at output channels of CFs corresponding to 0.5, 1, 2, 4, and 8 kHz to -20 dB FS, 40 μs condensation clicks. The *damping* in the system was set as 0.4, 0.5, and 0.7, respectively. The smaller damping corresponds to higher gain at all CFs. Figure 12F shows the calculated *Q*_*ERB*_ under different damping factors. The smaller *Q*_*ERB*_ corresponds to higher damping, and at higher damping (0.5 and 0.7), *Q*_*ERB*_ is higher at moderate CFs than lower and higher CFs.

The relation between dB FS and Sound Pressure Level, expressed in dB SPL, depends on the *damping* set-point used in the CAR-FAC model [*r*_1_ in Equation (10)]. Comparing the peak gain at moderate frequencies (1, 2, and 4 kHz) with the measured biological cochlea frequency response in Figure 1, we can see that using a *damping* factor of 0.4, the -20 dB FS input has ~60 dB peak gain, which fits the 30 dB SPL input intensity curve in Figure 1. Accordingly, at 0.5 *damping*, the -20 dB FS corresponds to 60 dB SPL, and at 0.7 *damping*, the -20 dB FS corresponds to 70 dB SPL.

**Figure 13:**
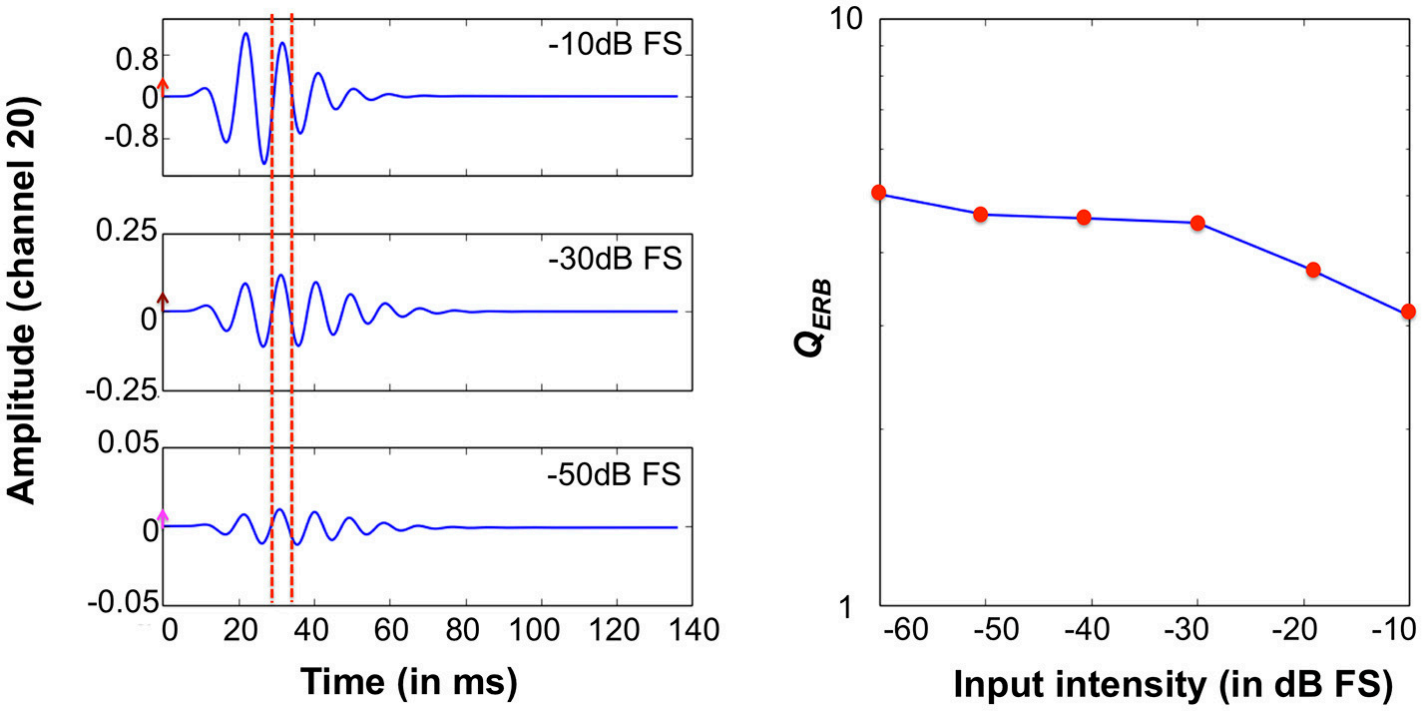
System impulse responses at the 1 kHz CF channel to -50 dB FS, -30 dB FS, -10 dB FS clicks. The arrows mark the amplitude of clicks. The red dashed lines mark two consecutive impulse response zero-crossings (**Left**). 1 kHz *Q*_*ERB*_ factors derived from impulse responses at relative intensities from -60 dB FS and -10 dB FS in steps of 10 dB FS (**Right**).

We also investigated the system's impulse response characteristics in the time domain and the intensity dependence of the *Q*_*ERB*_ factors. Figure 13 (Left) shows the CAR-FAC impulse responses at CFs corresponding to 1 kHz to a condensation click with -50 dB FS, -30 dB FS, and -10 dB FS intensity respectively. It shows the CAR-FAC filter characteristic that the shape and the amplitude of the impulse responses varied while the zero-crossing timing remains the same across the stimulus levels. Figure 13 (Right) shows the calculated *Q*_*ERB*_ factor for clicks with intensities between -60 dB FS and -10 dB FS in steps of 10 dB FS at the CF corresponding to 1 kHz. The *Q*_*ERB*_ factor decreases as the stimulus intensity increases. The sharpness of the frequency response thus decreases as the stimulus intensity increases.

### DIHC model output

To investigate the DIHC characteristics, we measured the DIHC response to tones. In order to present stimuli with same amplitude to the DIHC, we made use of the linearity of the CAR: we switched off the FAC function, leaving the CAR amplifying the input tones linearly. Firstly, we presented 0.5, 1, and 4 kHz tones to the system, and measured the CAR output at channels with CFs corresponding to each of those tones. We adjusted each tone's amplitude to make sure the CAR output at the corresponding channel had the same amplitude of 2.28 dB FS. Next, we used the adjusted tones as the input to the system and measured the DIHC output in response to those tones with the same CAR output amplitude at the corresponding CFs (Gmel et al., 2011).

**Figure 14:**
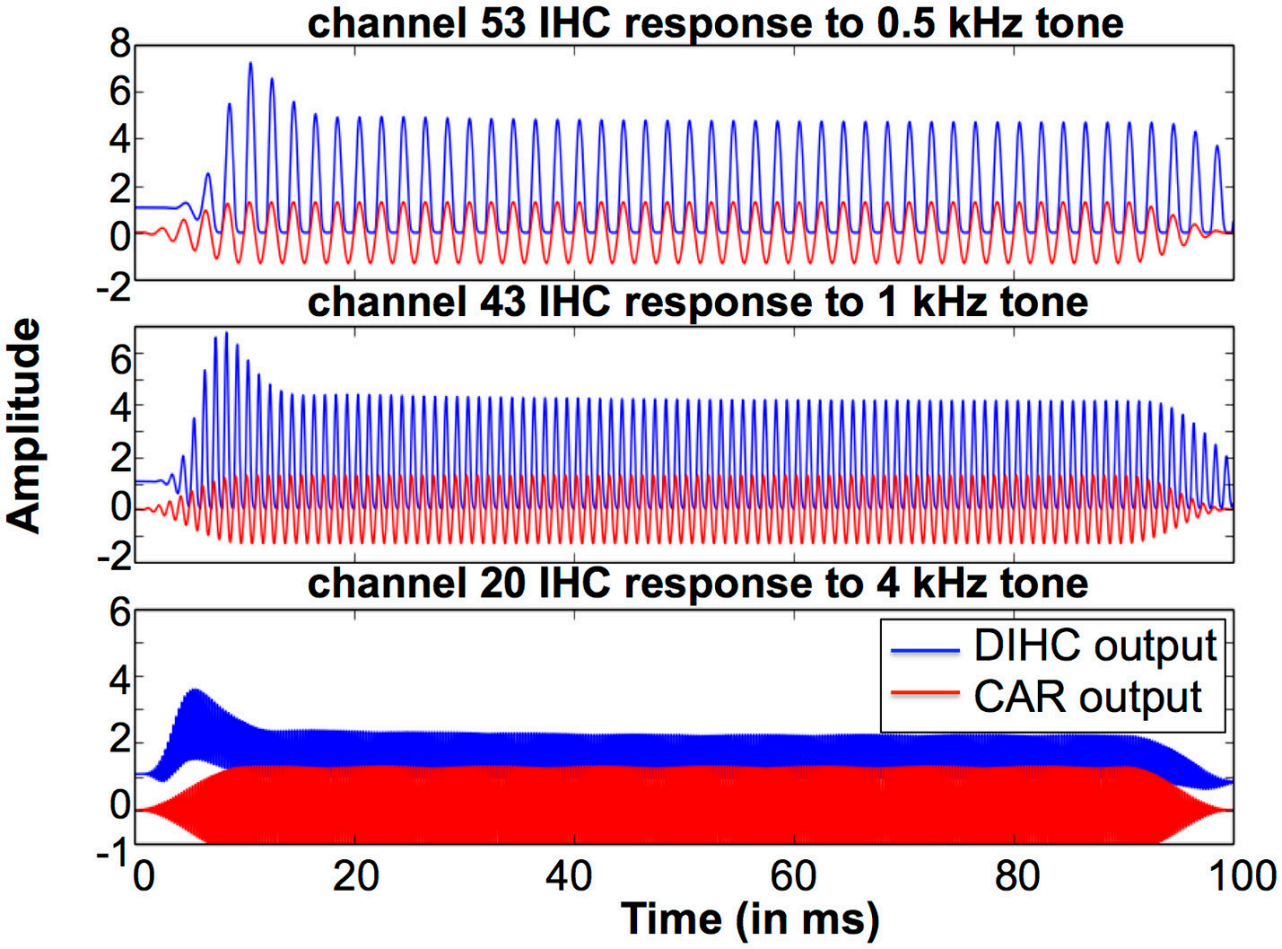
DIHC output and CAR output in response to 100 ms tones of 0.5, 1, and 4 kHz at the channels of CFs corresponding to those tones.

Figure 14 shows the DIHC output in response to 100 ms tones of 0.5, 1, and 4 kHz (squared-cosine rise and decay time of 10 ms). The DIHC detects and amplifies input signal onset well. For lower frequencies, e.g., 0.5 kHz, the DIHC output shows little DC offset and follows the sinusoidal curve of the input. As the input frequency is increased, the DIHC shows higher offset and reduced gain.

## Discussions

This paper presents a fully digital implementation of the CAR-FAC cochlear model. We use time-multiplexing and pipeline parallelizing techniques to implement a 70-channel real time CAR-FAC system at 44.1 kHz on a Cyclone V FPGA board. We measured the system responses to a set of stimuli such as pure tones and condensation clicks and analyzed the CAR-FAC nonlinear growth characteristics, excitation patterns, frequency selectivity and impulse response. We investigated the CAR-FAC *Q* tuning effects thought the damping factor tuning in Equation (10). Additionally, we measured the DIHC model responses to tones.

Here, we compare the system with prior silicon cochleae with respect to architecture, channel number, frequency range, input range, *Q* tuning, and power consumption, as shown in Table 2 (Fragniere, 2005; Sarpeshkar et al., 2005; Wen and Boahen, 2006; Yang et al., 2015, 2016). We use a power analysis tool, PowerPlay, provided by Altera to estimate the power consumption of the system on FPGA, since a direct measurement of the power consumption on the FPGA board is not possible for this development kit. Table 2 reports the estimated FPGA chip power consumption by PowerPlay based on its default settings. The CAR-FAC system shows a wide input frequency range and dynamic range, and a small *Q* tuning range. The power consumption of the whole FPGA board is high compared to other analog silicon cochleae. However, this fully digital system is stable, scalable, and easy to use. Additionally, it shows an outstanding agreement with the biological data recordings and an improved signal to noise ratio (SNR) (Saremi et al., 2016). It is thus able to provide an excellent input hardware stage to more complex machine hearing tasks such as sound localization, sound segregation, speech recognition, and so on.

**Table 2:** Comparison with prior silicon cochleae.

	**This work**	**Yang et al. (2016)**	**Yang et al. (2015)**	**Wen and Boahen (2006)**	**Sarpeshkar et al. (2005)**	**Fragniere (2005)**
Architecture	Cascade	Parallel	Parallel	Active coupling	Parallel	Passive coupling
Channel number	70 × 3^a^	64 × 2	16	360	16	100
Frequency range	up to 22.05 k Hz	8–20 k Hz	N/A	210–14 k Hz	100–5 k Hz	200–20 k Hz
Input range (dB)	70	73(including 18dB of the attenuator)	92	52	75(with AGC) 55(without AGC)	50
Power supply (V)	1.1	0.5	1.8	2.5	2.8	3.3
Power (*mW*)	1,260^b^	0.055	0.028	35.9	0.06	1.7
*Q* tuning	<10 (through *damping* tuning)	1.3-39 from channel 18	0.83-7	1.16 ± 0.92	<10	0.25–12

a *The FPGA ALM utilization is only 18% for one CAR-FAC module, so the system can be rescaled up to a maximum of 210 cochlear channels by implementing three CAR-FAC modules on the FPGA board*.

b *The power consumption of the CAR-FAC system is given as the whole FPGA chip power consumption including the PLLs, the DSPs, the RAMs, the IOs and the Logics, and the static power consumption of the whole chip is 240 mW*.

## Author contributions

YX, RW, and AvS: proposed the idea and designed the FPGA system; YX: recorded the data; YX, TH, RW, and AvS: evaluated and discussed the results; YX: wrote the manuscript. All authors discussed the results, commented on the manuscript and approved it for publication.

### Conflict of interest statement

The authors declare that the research was conducted in the absence of any commercial or financial relationships that could be construed as a potential conflict of interest.
